# Evaluating the Effectiveness of Return-to-Work Interventions for Individuals with Work-Related Mental Health Conditions: A Systematic Review and Meta-Analysis

**DOI:** 10.3390/healthcare11101403

**Published:** 2023-05-12

**Authors:** Behdin Nowrouzi-Kia, Pablo Garrido, Basem Gohar, Amin Yazdani, Vijay Kumar Chattu, Ali Bani-Fatemi, Aaron Howe, Andrea Duncan, Maria Paz Riquelme, Faizah Abdullah, Sharan Jaswal, Joyce Lo, Yusra Fayyaz, Bushra Alam

**Affiliations:** 1 Department of Occupational Science and Occupational Therapy, Temerty Faculty of Medicine, University of Toronto, 500 University Avenue, Toronto, ON M5G 1V7, Canada; 2 Krembil Research Institute, University Health Network, 60 Leonard Ave, Toronto, ON M5T 0S8, Canada; 3 Centre for Research in Occupational Safety & Health, Laurentian University, 935 Ramsey Lake Rd, Sudbury, ON P3E 2C6, Canada; 4 Escuela de Psicología, Universidad de Valparaíso, Hontaneda 2653, Valparaíso 2341369, Chile; 5 OH&S Salud Ocupacional, Av. Salvador 149 Of 811, Santiago 7500710, Chile; 6 Department of Population Medicine, University of Guelph, 50 Stone Road East, Guelph, ON N1G 2W1, Canada; 7 Canadian Institute for Safety, Wellness, and Performance, School of Business, Conestoga College Institute of Technology and Advanced Learning, 299 Doon Valley Drive, Kitchener, ON N2G 4M4, Canada; 8 Department of Community Medicine, Faculty of Medicine, Datta Meghe Institute of Medical Sciences, Wardha 442107, India; 9 Center for Transdisciplinary Research, Saveetha Dental College, Saveetha Institute of Medical and Technical Sciences, Saveetha University, Chennai 600077, India

**Keywords:** workplace, mental health, return to work, intervention, systematic review, meta-analysis

## Abstract

The workplace is a vital setting to support positive mental health. Mental health conditions in the workforce contribute to decreased work engagement and participation. There is existing literature on return-to-work (RTW) interventions for individuals with work-related mental health conditions, however, there lacks consensus on their effectiveness. Therefore, the primary aim of this systematic review was to synthesize the literature and evaluate the effectiveness of return-to-work interventions on return-to-work rates, quality of life, and psychological wellbeing for individuals with work-related mental health conditions. Selected articles were organized and identified using the Preferred Reporting Items for Systematic Reviews and Meta-analyses (PRISMA) guidelines and the Population/Intervention/Comparison/Outcome (PICO) framework. Quality assessment of the included studies was completed using the Critical Appraisal Skills Programme randomized controlled trials checklist and the Joanna Briggs Institute quasi-experimental studies checklist. A random effects meta-analysis model was performed using DerSimonian-Laird weighting to calculate standard mean difference and risk ratios to assess the impact of RTW interventions on return-to-work rates, absenteeism, stress symptoms, depression symptoms, and quality of life. A total of 28 out of 26,153 articles met the inclusion criteria. Diagnoses for participants in the studies ranged from work-related stress to work-related PTSD following exposure to a psychologically traumatizing event in the workplace. No significant differences were found for the meta-analyses examining return-to-work rates, absenteeism, depression, stress, and quality of life. The most effective interventions were found to be a multi-domain intervention (67% of participants RTW full time) and a health-focused intervention (85% RTW rate). Future research may consider establishing effective interventions to develop programs or policies supporting the RTW of employees and promote mental well-being among employees experiencing work-related mental health conditions.

## 1. Introduction

Workplace mental health is an important public health issue that impacts mental well-being. With the growing number of workers experiencing mental health problems and significant productivity losses, it is crucial to address and accommodate for the employment of individuals with mental health issues. The economic cost of poor mental health in the workplace is enormous. Moreover, approximately 15% of the global working population is affected by a mental health condition [1]. It has been reported that depression is the leading cause of reported “disability” within the workplace and employees who suffer from depression are five times more likely to experience unemployment in comparison to employees who do not. Various countries around the world have reported that mental health concerns are a potential cause for numerous employees leaving their jobs. For instance, one of the mutual insurance providers in Chile found that work-related mental health conditions constituted more than 28,000 lost workdays and is one of the leading causes of “occupation-related accidents” [2]. Furthermore, work-related mental health conditions have increased exponentially by 84% in regions such as Chile [3]. Mental health issues are also prevalent in working populations in Canada [4]. Poor psychological working conditions, or the presence of job stressors, can also increase the risk of developing depression, anxiety, burnout, and experiencing work-related distress [5].

Mental health issues in the workforce contribute to decreased work engagement, such as sick leave or long-lasting work disability [6]. In Canada, mental health conditions constitute 30% of work disability benefit claims [7] and represent 70% of the total cost of disability claims [8]. According to Hendriks et al. (2015), chronic mental health conditions, such as anxiety or depression, were strong predictors of long-term work absenteeism [9]. To mitigate the individual, social, and economic impacts of decreased work engagement and absenteeism in workers with mental health conditions, there is a need for effective interventions that support return-to-work (RTW) after a duration of sick leave or disability. 

There has been growing interest in the literature on interventions that support RTW for individuals with mental health conditions. Although the primary goal of interventions is to facilitate successful RTW, there is little consensus about which intervention is deemed the most effective in supporting this goal [10]. Interventions that have been shown to support RTW include a psychological component, such as cognitive-behavioral treatment/therapy (CBT), psychotherapy, stress-reduction treatment, psychoeducation, and medication [11,12]. According to a systematic review and meta-analysis by Nigatu et al. (2016) that looked at the effectiveness of CBT-based RTW interventions for workers with common mental health disorders, it was found that the interventions examined did not improve RTW rates but reduced the number of sick-leave days compared to a control [11]. This finding is supported by a systematic review by Cullen et al. (2017), which found strong evidence to suggest that CBT-based interventions that did not include workplace modifications or service coordination components were not effective in supporting the return-to-work of those with mental health conditions. Rather, Cullen et al. (2017) suggests that the implementation of interventions with multiple domains, i.e., healthcare provision, work accommodation, and service coordination, can help improve work function and reduce lost time due to mental health conditions [13]. 

Currently, there is growing interest for evidence-based reviews about RTW interventions for individuals with common mental health disorders [11,14,15,16]. However, there is a gap in the literature focused on RTW for individuals with mental health issues of workplace origin. Therefore, the aim of this systematic review is to evaluate the effectiveness of RTW interventions that improve return-to-work rates for individuals with work-related mental health conditions. Effective RTW was defined using a variety of definitions specified in the studies including partial or gradual RTW to the same employer [17], beginning of sick leave/absenteeism to “modified” or partial RTW (same employer, different duties/tasks), full-time RTW [18,19,20] or “complete” RTW [21], returning to work while simultaneously receiving treatment [20], “having a job and not being on sick leave”, [22], increased work hours [19], part-time or full-time RTW (0–75% sick leave) [23], “gainful employment” [24], partial RTW to any extent, and full RTW (working regular hours continuously for 4 weeks) [25]. Predictors and factors potentially associated with RTW were also examined including absenteeism, stress symptoms, depression symptoms, and quality of life. Meta-analyses of the impact of these interventions were also conducted to evaluate the benefits of these interventions and additional outcomes of interest, such as depression symptoms, stress symptoms, as well as quality of life scores. 

## 2. Materials and Methods

### 2.1. Overview 

This systematic review and meta-analysis followed the Preferred Reporting Items for Systematic Reviews and Meta-analyses (PRISMA) guidelines for the identification of relevant studies. The developed search strategy was carried out using the following databases: Ovid Medline, Embase, CINAHL, APA PsycINFO, and Cochrane Review. The Population/Intervention/Comparison/Outcome (PICO) framework was used to structure, support, and refine our search strategy and research question. This systematic review has been registered with PROSPERO with the following ID: CRD42022363111. 

### 2.2. Search Strategy and Study Selection 

The search criteria were established with the support from a health research librarian, and all included studies were published in English or Spanish. There was no restriction on the publication date to ensure a comprehensive review of the literature, however the focus was on articles published after the year 2000 to provide findings and evidence that is generalizable to the current working environment. For the search terms utilized, please see Appendix A. Article selection was retrieved on 6 August 2021, and our approach complied with the requirements of each database. Two reviewers were involved in screening the articles. The articles were screened in stages. First, the reviewers screened the search findings by article title to determine the relevance to the study aim and the keywords searched. Next, the reviewers reviewed the abstract of the remaining articles after title screening to ensure that the studies reported the return-to-work outcomes of interest for this systematic review: return-to-work rates, absenteeism, stress, depression, and quality of life. The reviewers independently analyzed each title and abstract to exclude papers that also did not meet the inclusion and exclusion criteria, which is shown in Table 1. The full texts of the selected intervention studies were then retrieved and reviewed for suitability for the systematic review and meta-analysis. To achieve consensus, any disagreement about a study’s inclusion at any stage was resolved by the reviewing committee. While diagnostic criteria varied for the participants, the included studies were organized into six different diagnostic categories: work-related adjustment disorders (*n* = 3), work-related anxiety disorders (*n* = 1), work-related burnout (*n* = 2), work-related depression (*n* = 4), work-related PTSD (*n* = 2), and work-related stress (*n* = 16). However, many study participants had overlapping diagnoses and conditions. All studies generated from the literature search were stored in Covidence, a commercially available systematic review management website [26].

### 2.3. Critical Appraisal of Included Studies 

The critical appraisal of the included studies was completed using either the Critical Appraisal Skills Programme (CASP) checklist for randomized controlled trials or the Joanna Briggs Institute (JBI) checklist for the quasi-experimental studies. The Critical Appraisal Skills Programme (CASP) randomized controlled trial checklist includes 11 questions across 4 domains (Section A: Is the basic study design valid for a randomized controlled trial? Section B: Was the study methodologically sound? Section C: What are the results? Section D: Will the results help locally?) [27]. The Joanna Briggs Institute (JBI) critical appraisal tool for quasi-experimental studies consists of nine questions regarding the variables, participant groups, intervention/exposure, follow up, outcomes and the statistical analysis. The results of the JBI critical appraisal aids to interpret the results of a study [28]. 

Each study was independently assessed by two reviewers, who scored each study on its risk of bias. Any disagreements that arose were resolved by the first author. If no consensus was reached, the conflicts were then brought to the larger team to be resolved. The critical appraisal of the articles focused on determining if the included studies were methodologically sound and the risk of bias in each study. For both the CASP and JBI checklists, a score of +1 was assigned for a low risk of bias, a score of −1 for a high risk of bias, and a score of 0 for an unclear risk of bias for each checklist item. The scores for each criterion were then summed up for each study. The studies’ characteristics, including study design, quality assessment, and risk of bias scores, are shown in Table 2. Diagnosis specific critical appraisal averages and standard deviations are shown in Table 3. 

### 2.4. Data Extraction

Data was extracted from each article by one reviewer. A random sample of 25% of included articles (n = 7) was double-checked to confirm the accuracy of data extraction. Each reviewer used the Cochrane data extraction form for systematic reviews of interventions provided in the Cochrane handbook [46]. The extracted data included the study characteristics (type of study, participants, type of interventions, types of comparisons, aim of study, design), methodological characteristics (duration of intervention, details of the intervention), participants characteristics (description of sample, subgroupings, setting, inclusion, exclusion criteria, methods of recruitment, randomization technique, demographic variables, psychological diagnoses, and medical co-morbidities). Variables of interest were also extracted including percent or count of the population who experienced RTW, quality of life outcomes, absenteeism outcomes, depression outcomes, stress outcomes, and PTSD symptoms. 

### 2.5. Meta-Analysis 

Meta-analyses were performed using the data from 19 of the 28 selected articles using the Preferred Reporting Items for Systematic Review and Meta-Analysis (PRISMA) guidelines. Data was cleaned to organize variables and ensure that all observations were reported in a consistent numeric format. Risk ratios were calculated to evaluate the effectiveness of each RTW intervention. Standardized mean differences were calculated using DerSimonian–Laird weighting for the random-effects meta-analyses assessing the impact of the interventions on RTW rates, absenteeism, depression, stress, and quality of life. I^2^ statistic was tabulated as an evaluation of the between study variation, which is an indication of the study heterogeneity. The meta-analyses were performed using R 4.0.5 and the “metafor” package [47]. Forest plots were constructed for each meta-analysis as a visualization of the intervention effects using standardized mean difference and were fitted using a linear model framework.

### 2.6. Intervention Categorization

Using the intervention categorization methods from the Cullen et al. (2017) study, the interventions in this systematic review were classified and grouped into four large domains. For the purposes of this study, the four large domains were slightly modified. The four domains are: “Health-focused Interventions”, “Service Coordination Interventions”, “Work-modification Interventions”, and “Multi-domain Interventions”. In the Cullen et al. (2017) study, the “Health focused intervention domain” consisted of health directed interventions that were administered to patients in a work-related environment. Examples include CBT, psychological therapy, occupational therapy, etc. [13]. In this systematic review, many interventions were administered within a hospital/university setting and therefore all health-directed interventions despite the setting were included in this domain. “Service coordination interventions” were described as interventions that increase accessibility to and help facilitate the RTW process. This includes ameliorating the communication between an employee and their supervisor using “RTW plans, case management, education and training [13]”. In addition to interventions that helped facilitate RTW, our systematic review also included service coordination interventions that address workplace stressors or assisted in preventing sick-leave. “Work modification interventions” involve changes to the work environment and workplace conditions. For instance, adjusting work tasks or work hours, etc. [13]. No modifications were made to the attributes of this domain. Lastly, the “Multi-domain interventions” are interventions that incorporated two or more of the intervention domains [13]. For example, an intervention that incorporates both an occupational therapy component (health-focused intervention domain) and a convergence dialogue meeting between the employee and supervisor (service coordination intervention domain) would be considered a multi-domain intervention. A total of 11 studies in this systematic review examined health focused interventions (39%), 5 studies investigated service coordination interventions (18%) and 12 studies investigated multi-domain interventions (43%).

## 3. Results

### 3.1. Search Outcome

**Figure 1:**
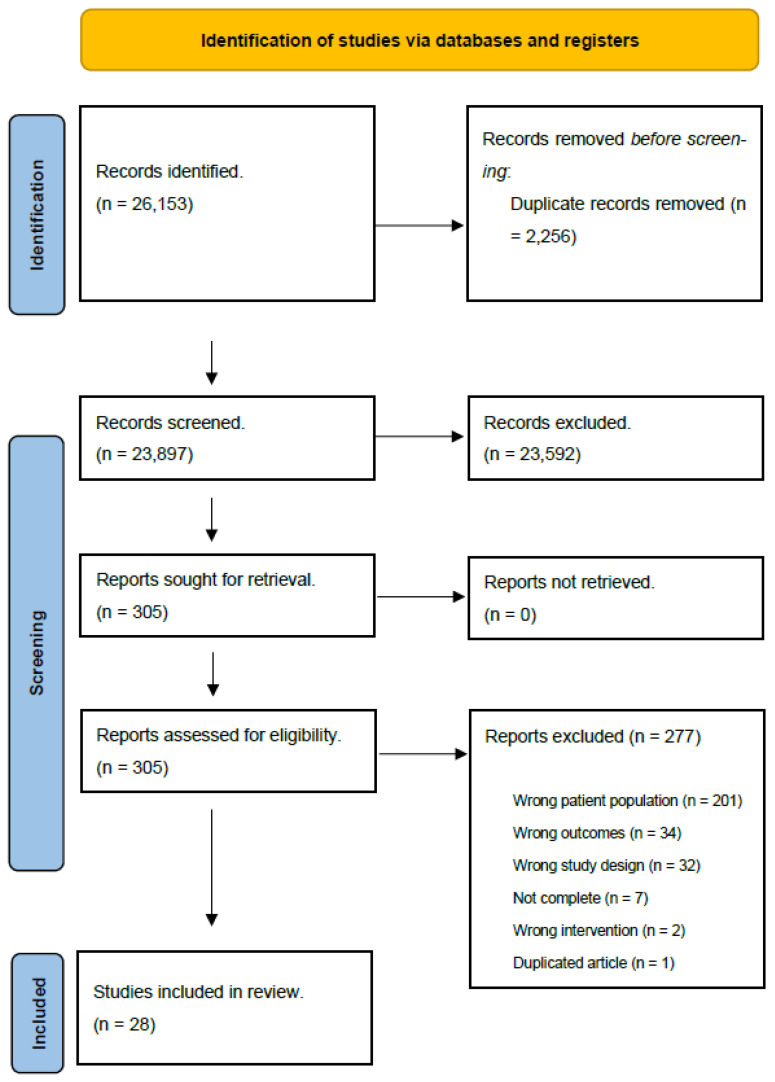
Preferred Reporting Items for Systematic Review and Meta-Analysis (PRISMA) diagram.

The initial search yielded 26,153 studies, of which 2256 duplicates were removed. Titles and abstracts were then screened by 2 reviewers, 23,592 of which were deemed to be irrelevant. Reviewers then analyzed 305 full-texts, 277 of which were excluded due to several reasons: a wrong patient population (*n* = 201), irrelevant outcomes (*n* = 34), the wrong study design (*n* = 32), being incomplete (*n* = 7), implementing a wrong intervention (*n* = 2), or being a duplication (*n* = 1). After full-text analysis, 28 studies were included in the review (Figure 1). These included both randomized controlled trials (*n* = 19) and quasi-experimental studies (*n* = 9). The included studies ranged in publication from 2000 to 2020, with most studies having been published in 2010 or later (*n* = 23). 

### 3.2. Critical Appraisal of Included Studies

The average CASP and JBI scores for articles are organized by diagnostic criteria in Table 3. For categories that contained only one study that used the CASP or JBI scale, the standard deviation is listed as 0. For the RCTs, the CASP score ranged from 1 to 10 with a mean of 7.53 (standard deviation [SD] = 2.34), thus indicating that the average risk of bias for the RCTs included in this study was relatively low and that they had an appropriate degree of reliability and quality. For the quasi-experimental studies, the JBI score ranged from 3 to 9 with a mean of 6.11 (SD = 2.15) indicating a medium/moderate level of reliability and quality and a mediocre risk of bias.

### 3.3. Interventions

Diagnoses for participants in the studies were diverse and ranged from work-related stress to work-related PTSD following exposure to a psychologically traumatizing event in the workplace. The systematic review found several types of interventions used to support the return of employees with work-related mental health conditions to the workplace, including convergence dialogue meetings between the employee and supervisor and psycho-educative internet programs. The health focused interventions identified in this study include CBT (*n* = 4) [21,31,33,36], psychotherapy (*n* = 5) [20,31,32,33,34,36,37,39], and occupational therapy (*n* = 2) [35,38] based interventions. The service coordination interventions include a combination of convergence dialogue meetings (CDM) between the employee and supervisor and questionnaires, interviews and seminars for both employee and supervisor (*n* = 2) [17,23], a solution-based intervention comprising of interviews between employee and supervisor (*n* = 1) [25], a “Workplace Mental Health Promotion Program” (comprised of: coping mechanisms, stress management and relaxation techniques, sharing personal experiences with work-related stress factors, brochure with intervention instructions, daily practice of techniques) (*n* = 1) [35] and a “psychodynamic online intervention” (*n* = 1) [45]. Most of the multi-domain interventions encompassed elements of two domains, the health-focused intervention domain and the service coordination intervention domain (*n* = 10) [18,24,29,30,40,41,42,43,48,49]. Two studies included interventions that incorporated elements from all three domains (the first study examined ‘a multi-disciplinary stress treatment program’ involving a series of steps; identifying stress factors, examining the participants’ (employees) workloads, exposure to various stress management techniques (e.g., exercise and ‘relaxation techniques’), providing a stress manual and connecting employees with their respective supervisors regarding work changes to help alleviate work-related stress [22]. In the second article, researchers examined the effect of a multi-disciplinary intervention that involved a combination of ‘work-related psychotherapy’ and a ‘mindfulness-based stress reduction course’ within a group setting [19]). 

### 3.4. Meta-Analytic Findings

**Figure 2:**
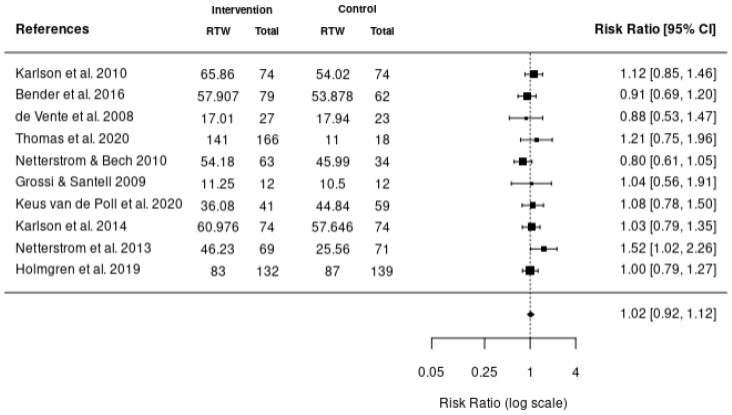
Forest plot of the effectiveness of RTW intervention studies on RTW rates [17,18,19,20,21,22,23,24,25,40].

**Figure 3:**
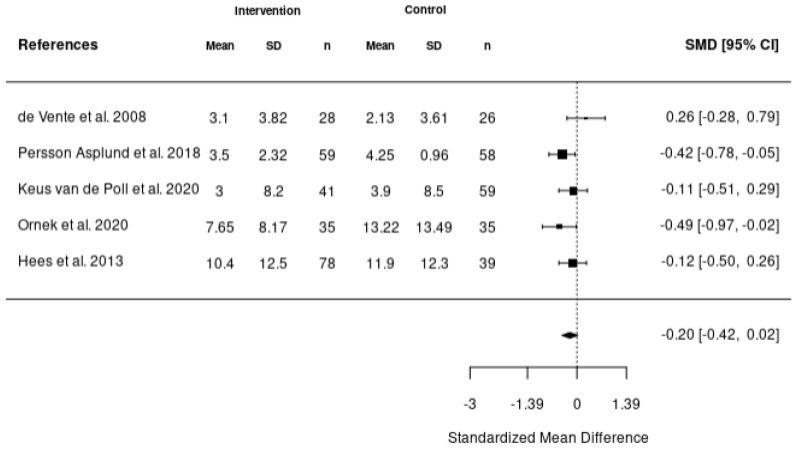
Forest plot of the impact of RTW interventions on absenteeism [21,25,35,44,49].

**Figure 4:**
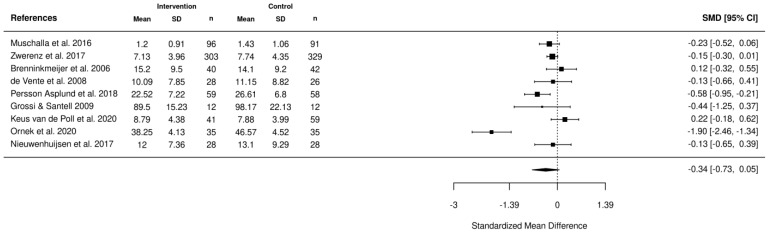
Forest plot of the impact of the RTW interventions on stress [1,21,24,25,36,41,45,48,49].

**Figure 5:**
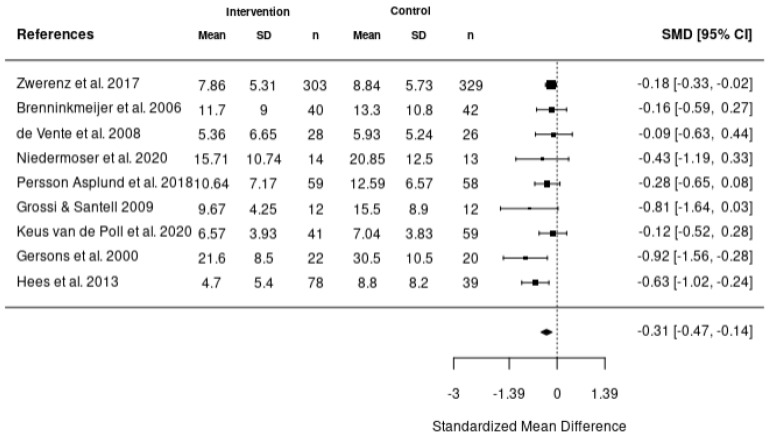
Forest plot of the impact of RTW interventions on depression [9,21,24,25,34,37,45,48,49].

**Figure 6:**
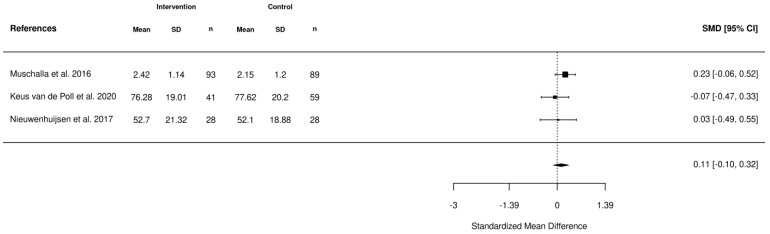
Forest plot of the impact of RTW interventions on quality of life [25,36,41].

Five meta-analyses were performed. The first meta-analysis investigated the impact of various interventions including health-focused, service coordination, and multi-domain on the return-to-work of employees using the data reported from 10 studies (Figure 2). In addition, meta-analyses were also completed to investigate the impact of these interventions on absenteeism (Figure 3), stress (Figure 4), depression (Figure 5), and quality of life (Figure 6). According to the meta-analyses completed, the interventions were marginally effective (95% CI, 1.02 [0.92, 1.12]) on the RTW rates of employees in comparison to the control groups (Figure 2). The most effective intervention for promoting RTW was a multi-domain intervention (a multi-disciplinary intervention incorporating aspects of all three domains) and a health-focused intervention (group psychotherapy + specialty mental health treatment program) [19,20]. Moreover, the intervention groups did not appear to have an overall improvement on absenteeism (95% CI, −0.2 [−0.42, 0.02]), stress symptoms (95% CI, −0.34 [−0.73, 0.05]) or depression symptoms (95% CI, −0.31 [−0.47, −0.14]) in comparison to the control. In contrast, the interventions appeared to have a slight improvement on quality of life in comparison to the control groups (95% CI, 0.11 [−0.1, 0.32]). There was no statistically significant difference in each of the meta-analyses performed.

## 4. Discussion

The objective of this systematic review was to evaluate the effectiveness of return-to-work (RTW) interventions aimed at promoting return-to-work for individuals with work-related mental health conditions. This systematic review and meta-analysis combined findings from studies and analyzed the impact of various interventions on RTW, PTSD symptoms, absenteeism levels, stress levels, depressions levels, and the participants’ quality of life. The systematic review identified several types of interventions used to support the RTW of employees experiencing work-related mental health conditions, each having a potentially different impact on the RTW process. These interventions included health focused interventions (CBT-based interventions,) service coordination interventions, (work-related or workplace-oriented interventions, convergence dialogue meetings, etc.) and multi-domain interventions (multi-disciplinary interventions, the “Best practice Intervention; incorporates both psychological and rehabilitative components and consulting a general practitioner). Our study found that quality of life and RTW rates were positively influenced by health focused, service coordination, and multi-domain interventions. Differently, absenteeism, stress, and depression levels did not appear to significantly improve by any of the intervention domains.

In a previous study a combined intervention including Cognitive Behavioral Therapy (CBT) and a “Return-to-work” module was evaluated [50]. This combined intervention examined in the study uses CBT methods (addressing negative/unwanted thoughts, behavioral changes, etc.) and revolves around work-place conflict and implementing the course of return-to-work. The study resulted in 55% of the participants who were experiencing work-related depression returning to work and 25% finding a new place of employment [50]. In comparison, of the studies included in this systematic review that investigated CBT related interventions, one study found no conclusive results regarding the RTW of employees [21]. However, two studies did seem to find a similar effect of CBT interventions on the RTW rates of employees as in previous studies. Employees with work-anxiety within the intervention group returned from sick leave approximately five weeks earlier than those in the control group [36]. The second study identified that the “work-focused CBT intervention” group returned to work four weeks earlier than the control group [43].

The findings of Cullen et al. (2017) suggested that out of the four identified domains, the multi-domain interventions were the most effective for those with mental health conditions as they returned to work earlier [13]. Moreover, a similar study to the current systematic review by Ansoleaga et al. (2015) suggested that a multidisciplinary treatment approach may improve the return-to-work process and raises the fact that the intervention must incorporate aspects of the workplace (facilitating the return to work), of the individual/employee (e.g., positive outlook on work) as well as the treatment itself (e.g., psychotherapy with a focus on all persons involved (supervisor, patient, family, etc.)) [51]. Based on the studies found in this systematic review, a multi-domain intervention combining aspects of all three domains; a combination of psychotherapy (health), a stress reduction program (service coordination), and assessing participant workloads and enforcing task modifications while communicating with respective employers (work modification) appeared to significantly improve the RTW rates of employees (67% of employees returned to full time work after exposure to the treatment), which aligned with previous research [19]. 

Other studies have also recommended that communicating with the workplace is crucial for patients with workplace related mental health conditions [51], which some of the interventions included in this systematic review revolve around [17,22,25,34,35]. 

Regarding the other factors that were examined in this systematic review including quality of life, stress, depression, and absenteeism levels, an important aspect to consider is that although these factors can impact RTW rates, RTW interventions are not directly intended to improve these factors. These interventions can potentially mediate or moderate these factors, however they are designed to primarily facilitate the RTW process and enable employees to remain employed and at work. 

It has been acknowledged that there is a paucity of studies that have been able to identify the most effective or profoundly effective return-to-work interventions for those that have mental disorders originating from the workplace. This study offers an evaluation of the various return-to-work interventions for those who have experienced work-related mental health conditions (depression, stress, etc.) and not only common mental disorders. This study offers a valuable contribution by conducting a meta-analysis that establishes the efficacy of each of these interventions on various work-related mental health illnesses such as stress levels and quality of life in attempts of finding the most effective intervention. This in turn could be integrated into return-to-work policies. Moreover, RTW interventions can be a tool to improve multidisciplinary mental health interventions. For mental health practitioners, this review demonstrates the need to integrate evidence to support RTW with clinical judgement. This is particularly relevant given the increased attention given to workplace mental health outcomes. Furthermore, the foundational role of workplaces plays a role in supporting the mental health for workers and how the COVID-19 pandemic may have affected that relationship [52].

### 4.1. Implications 

Work-related mental health conditions are not prioritized enough, which increases the stigma against them within the workplace. To allow a safe space for employees to seek support, employers need to show that they will prioritize and advocate for their needs by implementing interventions for workplace mental health. This will also increase awareness amongst other employees without mental health conditions. Studies such as the current systematic review and meta-analysis can bring awareness and acknowledgement towards work-related mental health conditions and the importance of addressing this issue within the workplace. Moreover, work-related mental health conditions pose a significant economic burden on high-income nations such as Great Britain, causing governments to dispense up to GBP 26 billion annually [53]. One of the most significant contributors to these costs is the “loss of productivity” due to work-related sickness leave [54]. Studies included in this current systematic review offer brief discussions of the cost-effectiveness of interventions and costs associated with sickness leave [18,29,38]. Thus, this study will also provide brief insight into interventions that may potentially address these significant losses by decreasing the number of employees on sick leave due to work-related events. 

### 4.2. Limitations

In general, one of the main limitations of a systematic review is that it cannot avoid or correct the biases that already exist in the studies included in the review. For instance, during the critical appraisal, Netterstrom et al. (2013) [19] scored ‘one’ on the CASP scale, indicating that it has a high risk of bias that the systematic review could not eliminate. Another potential source of bias includes selection bias, which occurs when a systematic review fails to uncover all available and relevant data. This is a possibility as the authors excluded studies based on an “irrelevant outcome”, and there is a possibility that we have inadvertently omitted studies investigating statistically significant interventions while exploring “relevant outcomes” as a secondary outcome without evidently highlighting them throughout the entire study. Hence, the possibility of resulting in statistically insignificant results thereby presents another limitation of this study. Additionally, another limitation could be that since many of the studies were completed in Europe using European samples, external validity to other countries may be limited [55]. To elaborate, in different countries, employers may have varying systems and accommodations for employees returning to work and in turn this may have an impact on how quickly employees return to work. These same work regulations may not exist in other countries and therefore, the results cannot be applied to them in the same way which may compromise the external validity of the study. In addition, there are certain studies included in this systematic review that had a significantly higher Risk Ratios (RR) than the other studies included in the meta-analyses. For instance, the study by Netterstrom et al., 2013, had a risk ratio of 1.52 in the meta-analysis investigating the impact of interventions on RTW rates. In comparison to other studies included in that meta-analysis, having RRs ranging from 0.80 to 1.21, a risk ratio of 1.52 appears to be an outlier, potentially skewing the results to appear to influence RTW rates [19]. Moreover, as previously stated, the Netterstrom et al., 2013 [19] study had a high risk of bias, which may explain the substantially higher RR in comparison to the other studies.

## 5. Conclusions

The workplace is a vital setting to support and maintain positive mental health, yet work-related mental health conditions have become a greater concern for employees in a variety of industries. Establishing effective interventions may help workplaces develop programs and policies supporting the return-to-work of employees, as well as promote a better mental state through the reduction of depression and stress symptoms while also enhancing the quality of life. Regarding RTW, according to the meta-analyses the most effective interventions appear to be a multi-domain intervention (a multi-disciplinary intervention incorporating aspects of all three domains) and a health-focused intervention (group psychotherapy + specialty mental health treatment program) [19,20].

Further recommendations for research include further analysis of the available interventions supporting RTW for employees as most of the data displayed marginally effective interventions or insignificant results. Although this does not indicate that the interventions are ineffective, no credible proof has however been found regarding the effect of the interventions on the different outcomes. More statistically significant data could provide more conclusive results regarding the most effective intervention. For future studies, to achieve significant results, it is important to eliminate any potential confounding factors within the included studies that may have impacted the results. It is also important to investigate studies with higher-than-average risks of bias thoroughly to potentially avoid including such studies in the analysis to avoid skewing the results. In addition, it may be worthwhile to investigate the difference in results between studies such as this one and studies that have found statistically significant results to identify any inconsistencies in study methods, etc. Moreover, to further validate the favorable outcomes of interventions, future research may consider examining how interventions will be implemented as well as the tenability of these interventions [56]. To elaborate, to increase the effectiveness of interventions, aspects improving the sustainability of interventions is crucial to consider. Some of these aspects include providing sufficient resources so stakeholders remain “continuously committed” to supporting the implementation of the intervention. Another aspect to consider is that the interventions should contain a flexible process so they can be applied even when the employees’ situations change [55]. Once research has concluded the most effective intervention, it is recommended that this is implemented in workplaces to accommodate employees with workplace-related mental health conditions.

## Figures and Tables

**Table 1:** The inclusion and exclusion criteria used during article screening are listed.

Inclusion Criteria	Exclusion Criteria
▪ Language: English (or Spanish)	▪ Language: not English or Spanish
▪ Peer-reviewed research article, such as but not limited to: randomized control trials, cohort studies; emphasis on quantitative results) ▪ Mental health condition/issue that is work-related/workplace-related/originated due to work or from the workplace environment. ▪ Description of an intervention/method (e.g., clinical, psychological, work-based) focused on supporting employees return-to-work to their pre-absence workplace; reports on return-to-work outcomes ▪ Participants: over 18 years old (employees) ▪ Complete study (i.e., describes the results and conclusion)	▪ Any article that is a knowledge synthesis (e.g., literature review, systematic review, scoping review, rapid review, meta-analysis, meta-synthesis); any article that is an opinion piece/editorial/commentary piece. ▪ Qualitative studies were not included in this analysis ▪ Does not include a description of an intervention/method (e.g., clinical, psychological, work-based) focused on supporting employees return-to-work to their pre-absence workplace ▪ Does not report on return-to-work outcomes ▪ Participants: not over the age of 18 (e.g., children) ▪ Study is incomplete (i.e., it is a research protocol or a conference abstract)

**Table 2:** Characteristics of the included studies, organized by intervention type.

Author, Year, Country	Study Design; Sample Size	Diagnostic Criteria	Intervention and Domain Category	CASP Grade	JBI Grade
**CBT-based intervention**					
Brenninkmeijer et al., 2006, Netherlands [6]	RCT; *n* = 122	Work-Related Adjustment Disorder	Cognitive behavioral therapy (CBT) and Combined intervention and Multi-Domain	7	n/a
Dalgaard et al. 2017., Denmark [29]	RCT; *n* = 163	Work-Related Stress	Work-focused CBT intervention + “optional workplace intervention and Multi-Domain	10	n/a
**de Vente et al., 2008,** **Netherlands [21]**	RCT; *n* = 82	Work-Related Stress	Group and Individual CBT based stress management training (SMT) and Health-Focused	4	n/a
Glasscock et al. 2018., Denmark [30]	RCT; *n* = 137	Work-Related Stress	Stress Management Intervention-Individual CBT + Workplace Intervention and Multi-Domain	6	n/a
Willert et al. 2011., Denmark [31]	Randomized waitlist-controlled trial; *n* = 102	Work-Related Stress	Group-format cognitive behavioral stress management intervention and Health-Focused	9	n/a
**Health-focused intervention**					
Beck et al., 2015, Denmark [32]	Randomized waitlist-controlled trial; *n* = 20	Work-Related Stress	Guided Imagery and Music (GIM) Intervention and Health-Focused	8	n/a
Collins et al., 2020, Australia [33]	Pilot pre-post intervention design; *n* = 81	Work-Related Stress	App-based intervention called “Anchored” and Health-Focused	n/a	3
Gersons et al., 2000, Netherlands [34]	Randomized controlled clinical trial; *n* = 42	Work-Related PTSD	“Brief Eclectic Psychotherapy” and Health-Focused	8	n/a
Hees et al., 2013, Netherlands [35]	RCT; *n* = 117	Work-Related Depression	Adjuvant occupational therapy and Health-Focused	10	n/a
Muschalla et al., 2016, Germany [36]	cluster RCT; *n* = 345	Work-Related Anxiety Disorder	Work-anxiety coping group (WAG) and Recreational Group (RG) and Health-Focused	6	n/a
Niedermoser et al., 2020, Switzerland [37]	Randomized interventional pilot study; *n* = 27	Work-Related Depression	Work-related Interpersonal Psychotherapy (W-IPT) and Health-Focused	7	n/a
Schene et al., 2007, Netherlands [38]	RCT; *n* = 62	Work-Related Depression	Treatment as usual + Occupational Therapy and Health-Focused	10	n/a
Schramm et al., 2020, Germany [39]	Monocentric RCT; *n* = 28	Work-Related Depression	Work-related Interpersonal Psychotherapy (W-IPT) and Health-Focused	10	n/a
Thomas et al., 2020, USA [20]	Pilot program; *n* = 166	Work-Related Stress	Work Recovery Group (WRG) and Health-Focused	n/a	4
**Multi-domain intervention**					
Bender et al. 2016, Canada [18]	Pre-post intervention design; *n* = 141	Work-Related PTSD	Best Practice Intervention (BPI) and Multi-Domain	n/a	7
Grossi & Santell 2009, Sweden [24]	Quasi-experimental study; *n* = 24	Work-Related Stress	Stress Management Program and Multi-Domain	n/a	6
Holmgren et al., 2019, Sweden [40]	Two-armed RCT; *n* = 271	Work-Related Stress	Work Stress Questionnaire brief intervention + feedback from a general practitioner and Multi-Domain	7	n/a
Netterstrom & Bech 2010, Netherlands [22]	Prospective longitudinal study; *n* = 107	Work-Related Adjustment Disorder	Multi-disciplinary stress treatment program and Multi-Domain	n/a	7
Nieuwenhuijsen et al., 2017, Netherlands [41]	Randomized placebo-controlled trial; *n* = 96	Work-Related Stress	Coaching + light therapy and pulsed electromagnetic field (PEMF) therapy and Multi-Domain	7	n/a
Persson Asplund et al., 2018, Sweden [42]	RCT; *n* = 117	Work-Related Adjustment Disorder	Guided internet-based stress management (iSMI) and Multi-Domain	10	n/a
Rannard et al., 2014, United Kingdom [43]	Feasibility-controlled trial; *n* = 60	Work-Related Stress	Case-managed support received from general practitioners + workplace health advisers and Multi-Domain	n/a	4
**van de Leur et al., 2020, Sweden [44]**	Standardized clinical multimodal intervention; *n* = 393	Work-Related Burnout	Multimodal Intervention (MMI) and Multi-Domain	n/a	6
**Workplace Service** **Coordination Intervention**					
Karlson et al., 2010, Sweden [17]	Prospective controlled study; *n* = 148	Work-Related Stress	Workplace oriented Intervention and Service Coordination	n/a	9
Karlson et al., 2014, Sweden [23]	Follow-up analysis; *n* = 136	Work-Related Burnout	Workplace oriented Intervention and Service Coordination	n/a	9
**Keus van de Poll et al., 2020, Sweden [25]**	Two-armed RCT; *n* = 100	Work-Related Stress	Brief problem-solving intervention (PSI) and Service Coordination	8	n/a
Ornek et al., 2020, Turkey [45]	Pre-post non-equivalent control groups design; *n* = 70	Work-Related Stress	Workplace Mental Health Promotion Program based on Work-related stress model and Service Coordination	9	n/a
Zwerenz et al., 2017, Germany [46]	RCT; *n* = 664	Work-Related Stress	Transdiagnostic psychodynamic online intervention and Service Coordination	6	n/a
**Multi-domain and** **health-focused intervention**					
Netterstrom et al., 2013, Denmark [19]	Randomized waitlist-controlled trial; *n* = 198	Work-Related Stress	Multi-disciplinary stress treatment program and Multi-Domain and Health-Focused	1	n/a

**Table 3:** Diagnosis-specific critical appraisal averages and standard deviations.

Diagnosis	Sample Size	CASP Mean (SD)	JBI Mean (SD)
Work-Related Adjustment Disorder	3	8.500 (2.121)	7 (0.000)
Work-Related Anxiety Disorder	1	6.000 (0.000)	n/a (n/a)
Work-Related Burnout	2	n/a (n/a)	7.5 (2.121)
Work-Related Depression	4	9.250 (1.500)	n/a (n/a)
Work-Related PTSD	2	8.000 (0.000)	7 (0.000)
Work-Related Stress	16	6.818 (2.562)	5.2 (2.387)

## Data Availability

Available upon request from the corresponding author.

## Appendix A

### Appendix A.1. MEDLINE

((occupational/or work-related.mp. [mp = title, book title, abstract, original title, name of substance word, subject heading word, floating sub-heading word, keyword heading word, organism supplementary concept word, protocol supplementary concept word, rare disease supplementary concept word, unique identifier, synonyms, population supplementary concept word, anatomy supplementary concept word]) OR (work/or workplace/or job/) OR (career/or employment/or profession*/) AND ((exp Anxiety/or anxiety.mp. or exp Anxiety Disorders/) OR (exp Depression/or depression.mp.) OR (Trauma.mp.) OR (adjustment disorder.mp. or exp Adjustment Disorders/) OR (Post traumatic stress disorder.mp. or exp Stress Disorders, Post-Traumatic/) OR (PTSD.mp. or exp Stress Disorders, Post-Traumatic/) OR (stress.mp.) OR (exp Burnout, Psychological/or exp Burnout, Professional/or burnout.mp.) OR (mental health.mp. or exp Mental Health/)) AND ((exp Rehabilitation, Vocational/or exp Return to Work/or exp Employment/or RTW.mp.) OR (absenteeism.mp. or exp Absenteeism/) OR (quality of life.mp. or exp “Quality of Life”/)) AND (intervention.mp. OR (exp Workplace/or workplace accomodation*.mp.) OR (Cognitive behavio?ral therapy.mp. or exp Cognitive Behavioral Therapy/or CBT.mp.) OR (exp Mental Disorders/or exp Anxiety Disorders/or exp Psychotherapy/or exp Depression/or psychological therapy.mp. or exp Depressive Disorder/or exp Cognitive Behavioral Therapy/) OR (adjuvant occupational therapy.mp.) OR ((recreation and group therapy).mp. [mp = title, book title, abstract, original title, name of substance word, subject heading word, floating sub-heading word, keyword heading word, organism supplementary concept word, protocol supplementary concept word, rare disease supplementary concept word, unique identifier, synonyms, population supplementary concept word, anatomy supplementary concept word]) OR (convergent dialogue meetings/or CDM.mp. [mp = title, book title, abstract, original title, name of substance word, subject heading word, floating sub-heading word, keyword heading word, organism supplementary concept word, protocol supplementary concept word, rare disease supplementary concept word, unique identifier, synonyms, population supplementary concept word, anatomy supplementary concept word])) (limit to yr = “2000–2020”) (limit 29 to Spanish or to English language).

### Appendix A.2. APA PsycInfo

(((exp Occupational Stress/or exp Psychosocial Factors/or exp Working Conditions/or exp Anxiety/or exp Stress/) OR (employment.mp. OR exp Occupational Health/or work related.mp.)) AND ((workplace stressors.mp. [mp = title, abstract, heading word, table of contents, key concepts, original title, tests & measures, mesh word]) OR (exp Anxiety Disorders/or exp Anxiety/) OR exp “Depression (Emotion)”/OR (exp Occupational Stress/or exp Posttraumatic Stress Disorder/or stress.mp. or exp Posttraumatic Stress/or exp Stress/) OR (adjustment disorder.mp. or exp Adjustment Disorders/) OR (PTSD.mp. or exp Stress Disorders, Post-Traumatic/) OR burnout.mp. OR (mental health.mp. or exp Mental Health/) OR exp Mental Disorders/)) AND (((“return to work” or RTW or return-to-work).mp. [mp = title, abstract, heading word, table of contents, key concepts, original title, tests & measures, mesh word]) OR (exp Employee Absenteeism/or absenteeism.mp.) OR ((“quality of life” or QOL).mp. [mp = title, abstract, heading word, table of contents, key concepts, original title, tests & measures, mesh word]) OR (Return to Work/or exp Rehabilitation, Vocational/or exp Employment/or work modification.mp.) AND ((exp Workplace/or workplace accommodation*.mp.) OR (exp Workplace Intervention/or exp Intervention/) OR (Cognitive behavio?ral therapy.mp. or exp Cognitive Behavioral Therapy/or CBT.mp.) OR (exp Mental Disorders/or exp Anxiety Disorders/or exp Psychotherapy/or exp Depression/or psychological therapy.mp. or exp Depressive Disorder/or exp Cognitive Behavioral Therapy/) OR (occupational therapy.mp. or exp Occupational Therapy/)) (limited to (human and yr = “2000–2020”) (limited to Spanish or English language).

### Appendix A.3. CINAHL

((MH “Work Environment+”) OR (MH “Employer-Employee Relations+”) OR (MH “Stress, Occupational+”) OR (workplace or work or employment)) AND ((MH “Anxiety+”) OR (MH “Depression+”) OR trauma OR (MH “Stress Disorders, Post-Traumatic+”) OR (MH “Stress+”) OR (MH “Burnout, Professional”) OR (MH “Mental Health”)) AND ((RTW OR “Return to work” OR return-to-work OR “return to work”) OR (MH “Absenteeism”) OR (MH “Sick Leave”) OR (quality-of-life OR QOL)) AND (intervention OR (MH “Job Accommodation”) OR (“general practitioner” or GP)) (Limiters-Published Date: 20000101-20201231) (Narrow by Language: English).

### Appendix A.4. Cochrane

(MeSH descriptor: [Occupational Stress] explode all trees and with qualifier(s): [rehabilitation—RH]) OR ((“work-related”):ti,ab,kw) OR workplace) AND ((MeSH descriptor: [Anxiety] explode all trees and with qualifier(s): [therapy—TH, rehabilitation—RH]) OR (MeSH descriptor: [Depression] explode all trees and with qualifier(s): [therapy—TH, rehabilitation—RH]) OR (trauma):ti,ab,kw OR (MeSH descriptor: [Adjustment Disorders] explode all trees and with qualifier(s): [psychology—PX, rehabilitation—RH, therapy—TH]) OR (MeSH descriptor: [Burnout, Professional] explode all trees and with qualifier(s): [therapy—TH, rehabilitation—RH]) OR (mental health):ti, ab, kw OR (MeSH descriptor: [Stress Disorders, Post-Traumatic] explode all trees and with qualifier(s): [psychology—PX, rehabilitation—RH, therapy—TH]) OR stress) AND ((intervention):ti, ab, kw OR ((MeSH descriptor: [Return to Work] explode all trees) OR (MeSH descriptor: [Absenteeism] explode all trees) OR (MeSH descriptor: [Quality of Life] explode all trees))).

### Appendix A.5. EMBASE

((((((exp Occupational Health/or Occupational.mp. or exp Occupational Health Services/or exp Occupational Stress/or exp Occupational Therapy/) OR work related.mp. OR (employment.mp. or exp Employment/) OR profession*.mp.) AND (exp Anxiety/or anxiety.mp. or exp Anxiety Disorders/) OR (exp Depression/or depression.mp.) OR Trauma.mp. OR (adjustment disorder.mp. or exp Adjustment Disorders/) OR (PTSD.mp. or exp Stress Disorders, Post-Traumatic/) OR stress.mp. OR (exp Burnout, Psychological/or exp Burnout, Professional/or burnout.mp.) OR (mental health.mp. or exp Mental Health/)) AND ((exp Employment/or return to work.mp. or exp Return to Work/or exp Rehabilitation, Vocational/) OR (absenteeism.mp. or exp Absenteeism/) OR (quality of life.mp. or exp “Quality of Life”/) OR (exp Return to Work/or exp Employment/or work modification.mp.))) AND (intervention.mp.)) OR (“Brief Eclectic Psychotherapy” or BEP).mp. [mp = title, abstract, heading word, drug trade name, original title, device manufacturer, drug manufacturer, device trade name, keyword heading word, floating subheading word, candidate term word]) (limited to Spanish OR to English language) (limited to yr = “2000–2020”).
